# Bar-coding neurodegeneration: identifying subcellular effects of human neurodegenerative disease proteins using *Drosophila* leg neurons

**DOI:** 10.1242/dmm.029637

**Published:** 2017-08-01

**Authors:** Josefin Fernius, Annika Starkenberg, Stefan Thor

**Affiliations:** Department of Clinical and Experimental Medicine, Linkoping University, SE-581 85 Linkoping, Sweden

**Keywords:** Neurodegeneration, Protein toxicity, Cellular effects, Axon transport, Apoptosis

## Abstract

Genetic, biochemical and histological studies have identified a number of different proteins as key drivers of human neurodegenerative diseases. Although different proteins are typically involved in different diseases, there is also considerable overlap. Addressing disease protein dysfunction in an *in vivo* neuronal context is often time consuming and requires labor-intensive analysis of transgenic models. To facilitate the rapid, cellular analysis of disease protein dysfunction, we have developed a fruit fly (*Drosophila melanogaster*) adult leg neuron assay. We tested the robustness of 41 transgenic fluorescent reporters and identified a number that were readily detected in the legs and could report on different cellular events. To test these reporters, we expressed a number of human proteins involved in neurodegenerative disease, in both their mutated and wild-type versions, to address the effects on reporter expression and localization. We observed strikingly different effects of the different disease proteins upon the various reporters with, for example, Aβ^1-42^ being highly neurotoxic, tau, parkin and HTT^128Q^ affecting mitochondrial distribution, integrity or both, and Aβ^1-42^, tau, HTT^128Q^ and ATX1^82Q^ affecting the F-actin network. This study provides proof of concept for using the *Drosophila* adult leg for inexpensive and rapid analysis of cellular effects of neurodegenerative disease proteins in mature neurons.

## Abstract (precis)

**Summary:** Toxic effects of neurodegenerative disease proteins on neuronal function and morphology can be addressed using an array of transgenic fluorescent reporters in the adult *Drosophila* leg.

## INTRODUCTION

Neurodegenerative diseases (NDs) have increasingly been linked to dysfunction of specific proteins, often unique to one disease, e.g. amyloid precursor protein (APP) to Alzheimer's disease (AD), parkin (Park) to Parkinson's disease (PD), huntingtin (HTT) to Huntington's disease (HD), and superoxide dismutase (SOD1) to amyotrophic lateral sclerosis (ALS) (Kaur et al., 2016; Lill, 2016; Nopoulos, 2016; Selkoe and Hardy, 2016). Moreover, different ND proteins normally have distinct functions and subcellular locations, further supporting the notion of a certain degree of disease uniqueness. In contrast to this view of uniqueness, many ND proteins appear to cause neuronal dysfunction and degeneration by interfering with the same fundamental cellular processes [e.g. axonal transport, unfolded protein response (UPR), endoplasmic reticulum stress and autophagy], in addition to oxidative and mitochondrial homeostasis (Ross and Poirier, 2004; Han and Shi, 2016; Weishaupt et al., 2016; Ahmad et al., 2017; Islam, 2017; Krench and Littleton, 2017; Lin et al., 2017). One possible reason for this dichotomy, at least in part, stems from the fact that it has been challenging to elucidate the *in vivo* role of the wild-type proteins and the dysfunction of the disease variants. This is in part attributable to the slow progression of ND in mammalian model systems and to the difficulty with readily obtaining single-neuron cellular resolution in aging animals. Hence, the impact of ND proteins, normal or mutated, on different neuronal cellular events remains poorly understood.

Owing to the wide range of powerful genetic tools, relatively low maintenance costs and rapid generation time, the *Drosophila melanogaster* model system is being increasingly used to address various aspects of human ND (Bilen and Bonini, 2005; Gistelinck et al., 2012; Sun and Chen, 2015; West et al., 2015; Lewis and Smith, 2016). In line with mouse and animal cell culture studies, expression of mutated human ND proteins in *Drosophila* results in shortened lifespan, locomotor defects and apoptosis (Sang and Jackson, 2005; Lu and Vogel, 2009). By contrast, expression of wild-type versions of these human ND proteins typically has little or no effect. These, and many other observations, support the conclusion that *Drosophila* studies are valuable to reveal basic features of the ND process and, in particular, to shed light on highly evolutionarily conserved cellular processes. So far, the majority of these studies have relied on eye morphology (rough eye), larval dissections and immunohistochemistry, locomotor behavior and lifespan as read-outs of proteotoxic effects.

Recently, axonal processes in adult *Drosophila* legs and wings were pioneered as readily available preparations for assessing axonal degeneration (Neukomm et al., 2014; Sreedharan et al., 2015). Here, we develop this concept further and identify several transgenic reporter transgenes that are informative regarding the effect of ND proteins on neurons. To this end, we test the robustness and selectivity of 41 available fluorescent transgenic reporters in adult legs. We identify a number of reporters that are readily observable in adult legs and that report on different aspects of neuron biology. To address the usefulness of these reporters, we express a number of human ND proteins in leg neurons and observe the effects upon fluorescent reporter expression and localization. These include both normal and familial forms of amyloid beta (Aβ), tau, SOD1, α-synuclein (SNCA), HTT, ataxin-1 (ATX1) and Park (Feany and Bender, 2000; Warrick et al., 2005; Khurana et al., 2006; Kim et al., 2008; Romero et al., 2008; Watson et al., 2008; Jonson et al., 2015). We find strong and highly selective effects of the various ND proteins upon the fluorescent reporters, which support previous known roles of these ND proteins, but also indicate new effects. This study establishes adult *Drosophila* leg neurons as a powerful system for addressing the neuronal cell biological effects of ND proteins, in particular with respect to axon transport, mitochondrial homeostasis and the actin cytoskeleton.

## RESULTS

### Expression of human disease proteins in glutamatergic neurons causes reduced lifespan and mobility defects

During the last decade, *Drosophila melanogaster* has become widely used as a model for understanding human ND. To expand the phenotypic read-out for protein neurotoxicity *in vivo* in *Drosophila*, we aimed to develop a method in which age-dependent analysis of neurotoxicity is possible, using fly leg neurons and axons.

**Fig. 1.**
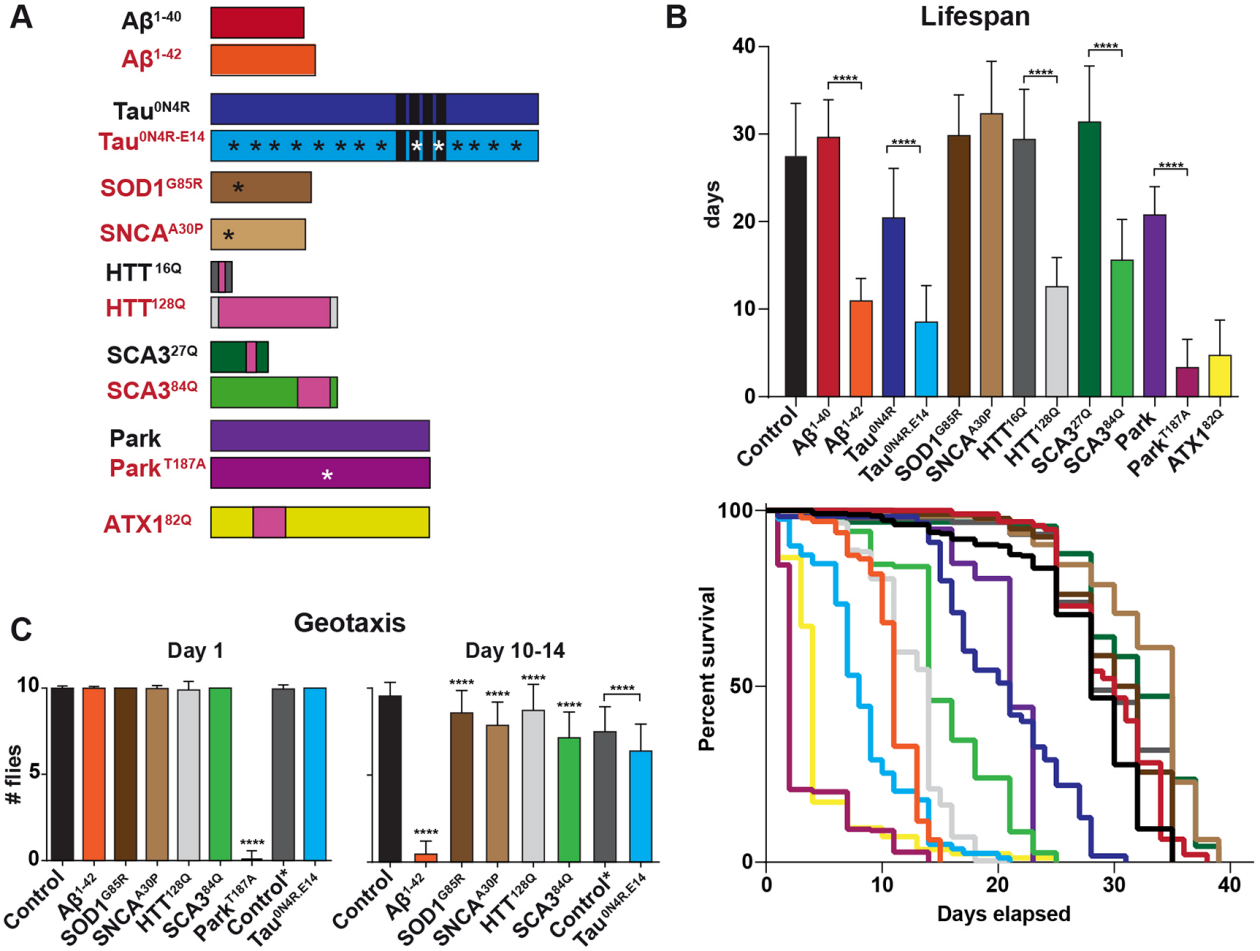
**Expression of human disease proteins in glutamatergic neurons results in reduced lifespan and mobility.** (A) Schematic representation of the disease proteins used, where the mutant protein variant is in red text. (B) Top, average lifespan of flies expressing human disease proteins using the *OK371-Gal4* driver, measured in days (mean+s.d.; ****P≤0.0001; Student's two-tailed *t*-test; *n*-values span from 48 to 324 control flies). Control flies are *OK371-Gal4*/*attP65B2*. Significant reduction in lifespan was observed for most mutant proteins, when compared with the wild-type proteins. However, SOD1^G85R^ and SNCA^A30P^ did not show reduced lifespan when compared with a control. Bottom, Kaplan–Meier survival curves. (C) Negative geotaxis assay showing locomotor activity. The graph indicates the average number of flies climbing to a 5 cm mark in 30 s, and the error bars indicate the s.d. All fly strains were compared with the control *OK371-Gal4*/*attP65B2*, and all tested flies were females, apart from control* (dark gray) and Tau^0N4R-E14^, which were males because low numbers of females hatched with Tau^0N4R-E14^. On day 1, only Park^T187A^ showed reduced locomotor activity, whereas on day 10-14, all flies showed an effect. Park^T187A^ and ATX1^82Q^ could not be tested at day 10-14 because of the short lifespan (mean+s.d.; ****P≤0.0001; Student's two-tailed *t*-test).

The *Drosophila* leg contains sensory neurons and their processes, in addition to the axonal processes and terminals from a number of leg motor neurons, all of which can be targeted by crossing *UAS* lines to the glutamatergic driver *OK371-Gal4* (Baek and Mann, 2009). Using this driver, we first addressed the toxicity of a number of human ND disease proteins, both wild-type and pathogenic/familial/dominant versions (herein referred to as mutant; Fig. 1A). Toxicity was addressed by crossing *UAS* transgenic lines to *OK371-Gal4* driver. To model AD, we made use of previously published *UAS* lines expressing amyloid beta peptides, *UAS-Aβ^1-40^* and *UAS-Aβ^1-42^* (Jonson et al., 2015). To address tau pathology, we used *UAS-Tau^0N4R^* and *UAS-Tau^0N4R-E14^* (a synthetic phospho-mimic and toxic version; Khurana et al., 2006). To model polyglutamine disease, we used *UAS-HTT^16Q^* and *UAS-HTT^128Q^* for HD (Romero et al., 2008); and *UAS-SCA3^27Q^*, *UAS-SCA3^84Q^* (SCA3 is also known as ATX3; Warrick et al., 2005) and *UAS-ATX1^82Q^* for ataxia (Fernandez-Funez et al., 2000). PD was modeled using wild-type *UAS-Parkin* (Park) and *UAS-PARK^T187A^* (Kim et al., 2008), in addition to mutated α-synuclein, *UAS-SNCA^A30P^* (Feany and Bender, 2000). ALS was modeled by expressing the *UAS-SOD1^G85R^* mutant (Watson et al., 2008).

Lifespan assay revealed that most of the mutant proteins and Aβ^1-42^ induced a significant reduction in lifespan when compared with control (*OK371-Gal4/attP65B2*) (Fig. 1B). However, the SOD1^G85R^ and SNCA^A30P^ mutants did not show any reduction in lifespan (Fig. 1B). Furthermore, the lifespan analysis revealed a significant difference between the wild-type and mutated versions of the proteins, with the mutated version giving rise to a significant reduction in lifespan (Fig. 1B). In some cases (Aβ^1-40^, SCA3^27Q^ and HTT^16Q^), expression of the wild-type version did not affect lifespan. By contrast, expression of wild-type Tau^0N4R^ and Park both gave rise to a significant reduction in lifespan, when compared with the control.

In order to obtain a physiological read-out of the effects of expression of these ND proteins, we next used a geotaxis assay to assess climbing ability, focusing on the most toxic protein mutants. Negative geotaxis was scored as the percentage of flies able to climb up the side of a vial in a set time. To address the effect of aging, geotaxis was tested both on adult day 1 and on day 10-14 (Fig. 1C). On day 1, expression of most of the mutant proteins did not give rise to reduced geotaxis, apart from Park^T187A^, which showed a severe defect in climbing ability (Fig. 1C). No further time point could be tested for Park^T187A^, because they did not live beyond day 2. Likewise, the day 10-14 time point could not be tested for ATX1^82Q^ because of its short lifespan. By day 10-14, all of the aged flies expressing human proteins and surviving to this later time point showed significantly reduced climbing ability (Fig. 1C). The results from lifespan and geotaxis assessments are in line with previous studies (Feany and Bender, 2000; Steinhilb et al., 2007; Romero et al., 2008; Watson et al., 2008; Gistelinck et al., 2012; Jonson et al., 2015), revealing mild or no effects for wild-type variants of these disease proteins, and stronger effects for most of the mutants.

### Survey of 41 fluorescent *UAS* marker lines identifies robust leg reporters

**Fig. 2.**
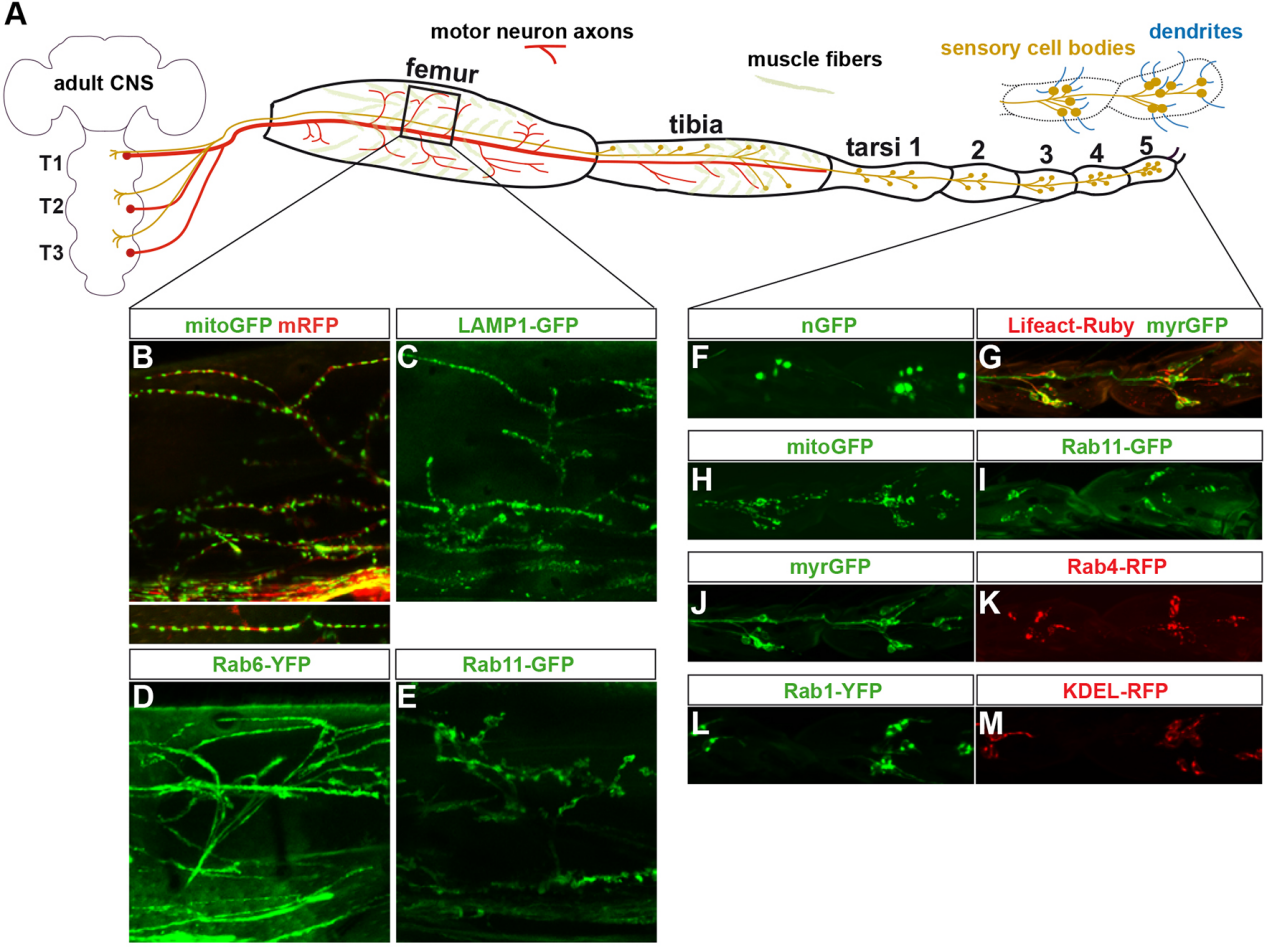
**Schematic diagram of the fly-leg model using *OK371-Gal4* driver and examples of fluorescent reporter proteins.** (A) Schematic representation of the fly leg and the nerves targeted by the *OK371-Gal4* driver. Red lines and dots depict motor neurons and their axonal terminals in the femur and tibia. Brown lines and dots depict sensory cells and their axonal projections. (B-E) Examples of projected confocal sections scanned through the femur, showing the indicated reporter proteins in axon terminals. *Z*-projection is shown below main panel in B. (F-M) Examples of projected confocal sections scanned through tarsi 4-5, showing the indicated reporter proteins in sensory neurons.

To identify markers that might be informative regarding the effects of ND proteins upon neuronal morphology and function, we screened 41 available *UAS* marker lines and tested their robustness in expression and their subcellular selectivity. These were tested in the nervous system of late larvae, using the *n-Syb-Gal4* driver, and in the adult leg neurons, using the *OK371-Gal4* driver (Fig. 2A; Table S1). We found that expression of many reporters was too weak to be detected readily by fluorescence microscopy. In particular, for adult leg neurons and axons/dendrites, the cuticle appears to reduce the signal and to create some degree of light scattering, which places high demands on the robustness and selectivity of the fluorescent markers. However, a subset of reporters showed robust expression and subcellular selectivity and were thus chosen for further study. These markers clearly distinguished different compartments of leg neurons, including the sensory neuron cell bodies, their dendrites and axons projecting into the central nervous system, and the axons and termini of motor neurons (Fig. 2A-M). These included mitoGFP, myristoylated monomeric-RFP (myr-mRFP), myrGFP, nuclearGFP (nGFP), Lifeact-Ruby, Rab1/4/6/11-RFP/GFP/YFP and LAMP1-GFP (Fig. 2B-M). For these markers, *UAS/OK317-Gal4* composite stocks were generated, and in some cases, two different *UAS* markers were combined with *OK371-Gal4*, in order to visualize two markers simultaneously (Fig. 2B,G).

### Analysis of sensory neuron survival using a nuclear-GFP marker

To monitor the effects of each ND protein upon various aspects of leg neuron biology, we expressed both wild-type and mutant forms of the human disease proteins under the control of the *OK317-Gal4/UAS* marker stocks.

**Fig. 3.**
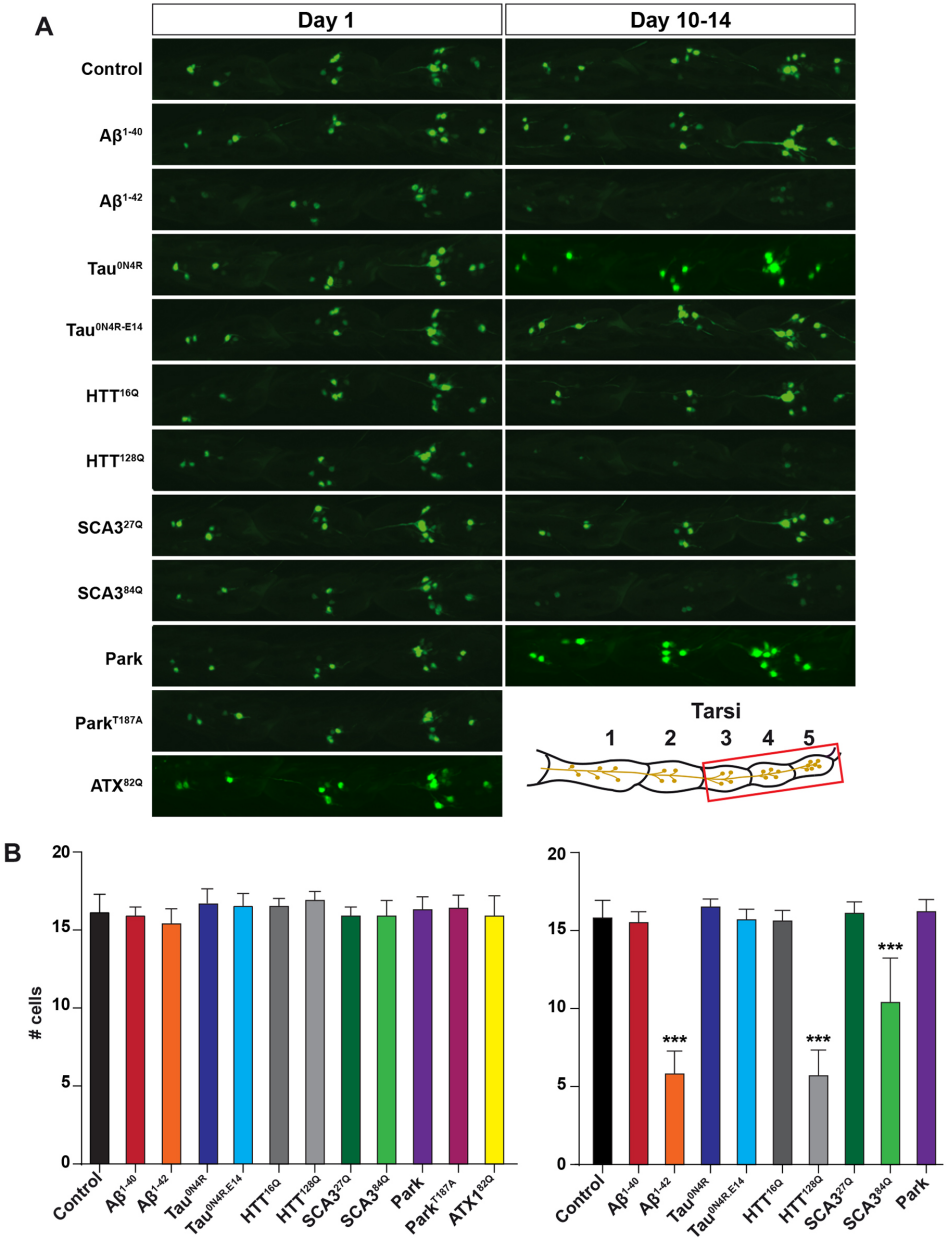
**Analysis of nuclear GFP marker as an indicator of cell viability.** (A) Representative images of projected confocal scans through tarsi 3-5, showing nGFP expression, for different genotypes, at day 1 and day 10-14. (B) Quantification of the presence of nGFP expression as a read-out of sensory cell numbers. Graphs show the average number of nGFP-expressing cells per fly leg. On day 1, no genotypes showed a reduced number of cells expressing nGFP. At day 10-14, Aβ^1-42^, HTT^128Q^ and SCA3^84Q^ all showed significantly fewer cells expressing nGFP (*n*=10 legs per genotype and age; mean+s.d.; ***P≤0.001; Student's two-tailed *t*-test, pair-wise against control).

First, we sought to analyze the cell survival of sensory neurons expressing disease proteins in tarsi 3-5, using the nGFP marker to visualize nuclei (Fig. 3). In controls at day 1, an average of 16 sensory cell nuclei was observed, with minimal variability (Fig. 3A-B). At day 1, none of the human proteins triggered any apparent loss of nGFP expression (Fig. 3A,B). At day 10-14, control tarsi still contained an average of 16 nGFP-expressing nuclei, whereas Aβ^1-42^, HTT^128Q^ and SCA3^84Q^ displayed significantly fewer expressing nuclei (Fig. 3A,B). Several proteins could not be assayed at these later time points because of lethality, including Park^T187A^ and ATX1^82Q^. In summary, there is no obvious loss of nGFP expression in any of the fly strains on day 1, but there is a significant loss of nGFP expression with age in flies expressing Aβ^1-42^, HTT^128Q^ and SCA3^84Q^, probably because the cells have died.

### Human disease proteins affect F-actin filaments in sensory neurons

Many studies have revealed that defects in the cytoskeleton constitute a common feature for many unrelated NDs. This includes not only the well-established links between ND and the stability of microtubule networks (Dubey et al., 2015), but also more recent findings that link ND with the integrity of the actin cytoskeleton (Eira et al., 2016). The actin cytoskeleton consists of actin monomers (G-actin) and flexible actin filaments (F-actin) and is crucial for neuronal shape, transport and cell motility (Kevenaar and Hoogenraad, 2015). Intriguingly, links have recently been proposed between ND and the integrity of actin filaments present in the axon initial segment (AIS; Sun et al., 2014; Tsushima et al., 2015).

To investigate any effects on F-actin when expressing ND proteins in the fly leg neurons, we used the Lifeact-Ruby marker, combined with myrGFP (mGFP) to label the entire neuronal cell. Lifeact marker fusions were previously generated by fusing the first 17 amino acids of the yeast Abp140 protein to fluorescent proteins, and these fusions robustly label the F-actin network in eukaryotic cells (Riedl et al., 2008).

**Fig. 4.**
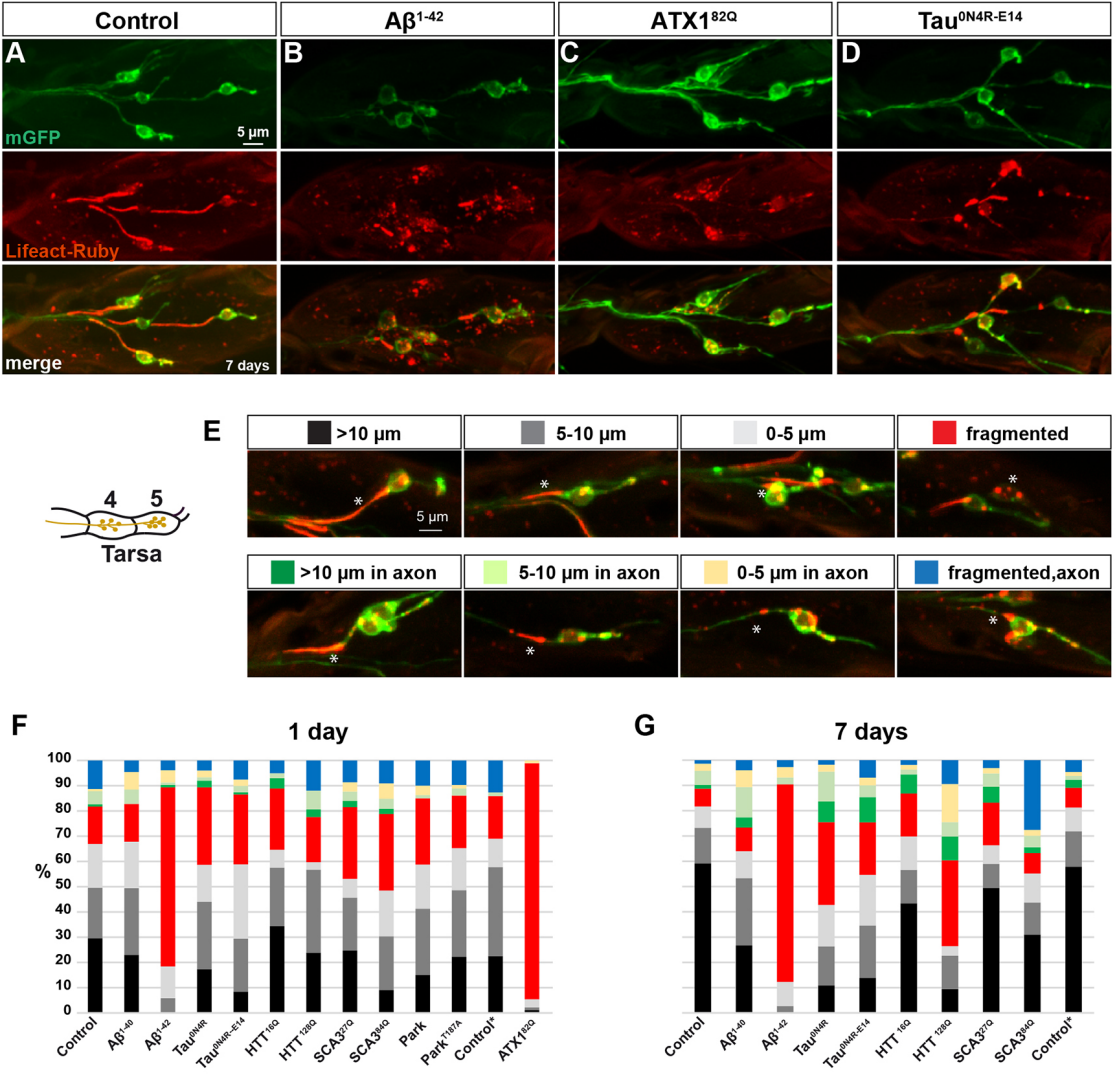
**Human neurodegenerative disease proteins affect F-actin in sensory cells.** (A-D) Control (*attP65B2*) and *UAS* disease protein lines were crossed to *OK371-Gal4*, *UAS-Lifeact-Ruby;UAS-mGFP* to reveal F-actin and cell outlines in sensory cell bodies in tarsi 4-5. A strong effect upon Lifeact-Ruby expression patterns, when compared with control, was observed in Aβ^1-42^, ATX1^82Q^ and Tau^0N4R-E14^. (E) Categories of Lifeact-Ruby patterns used for quantifying the effects seen with the different disease proteins (tarsus 5). Asterisk indicates the cell upon which each category is based. (F,G) Quantification of the Lifeact-Ruby pattern observed in the different disease strains, on day 1 and day 7. The graph shows the percentage of each Lifeact-Ruby category present in the sensory cells visualized in tarsi 4-5 (*n*= 67-115 cells for day 1, and *n*=53-130 cells for day 7). Flies were reared at +26°C and placed at +29°C for either 1 or 7 days, apart from control* and *UAS-ATX1^82Q^* flies, which were crossed at +20°C to enable viable offspring to hatch, after which they were transferred to +29°C.

Focusing on the leg sensory neurons in tarsi 4-5, at day 1 in control flies, we observe that Lifeact-Ruby robustly labels the initial axonal process, with close to 50% of cells showing a 1- to 20-µm-long Lifeact-Ruby-labeled process, and some 30% showing processes 10 µm or longer (Fig. 4A,E,F). However, there is some variability, even in the control, and the remaining 30% of neurons display 1- to 10-µm-long Lifeact-Ruby-labeled processes further away from the cell body, or fragmented staining in the axon or cell body (Fig. 4E,F). At day 7, in control flies, we observe an increase in the presence of the longer Lifeact-Ruby-labeled processes in the immediate axon to >70%, and reduction of the other categories (Fig. 4E,G). These experiments were conducted with flies reared at +26°C until eclosion, followed by overnight incubation at +29°C and analysis the next day (day 1) or on day 7. However, because ATX1^82Q^ expression resulted in few flies emerging, these crosses were reared at +20°C, after which they were transferred to +29°C overnight. Hence, matching control flies were also reared accordingly. These controls were not apparently different from controls reared at the higher temperature (Fig. 4F,G; asterisk).

Next, we turned to the human disease proteins, and again expressed both the wild-type and mutant protein variants in the leg neurons. Initially, we focused on day 1, a time point at which none of the human disease proteins displayed any obvious loss of sensory neurons (Fig. 3B), and therefore any effects observed would not merely reflect dying neurons. In addition, simultaneous labeling of cells with mGFP guided our analysis to cells with a robust mGFP signal. Strikingly, Lifeact-Ruby labeling revealed that several disease proteins caused profound effects, with ATX1^82Q^ and Aβ^1-42^ displaying a near-complete fragmentation of F-actin processes (Fig. 4B,F). In addition, Tau^0N4R^, Tau^0N4R-E14^ and SCA3^84Q^ displayed an apparent increase in fragmentation and reduction in the long Lifeact-Ruby axon processes (Fig. 4D,F). In general, the wild-type protein variants displayed fewer effects upon Lifeact-Ruby than the mutant ones (Fig. 4F). Surprisingly, Park^T187A^, in spite of its severe reduction of lifespan, with no flies surviving past day 2, and its severe geotaxis effects, did not show any dramatic effect on axon-process fragmentation reflected by an intact Lifeact-Ruby labeling (Fig. 4F). At day 7, the effects were even more pronounced, with severe fragmentation in Aβ^1-42^ and HTT^128Q^ flies (Fig. 4B,G). Interestingly, Tau^0N4R^ showed more fragmentation than Tau^0N4R-E14^ (Fig. 4G). In addition, Tau^0N4R^, Tau^0N4R-E14^, HTT^16Q^, HTT^128Q^ and SCA3^27Q^ displayed an increase in Lifeact-Ruby-labeled processes along the axons, a feature only observed in some 5-10% of cells in control flies (Fig. 4G). Surprisingly, this was not the case for SCA3^84Q^, which instead displayed an unparalleled increase in fragmented Lifeact-Ruby processes along the axon. Intriguingly, we find that the different disease proteins have diverse effects upon Lifeact-Ruby.

### Human disease proteins affect mitochondrial distribution in leg neurons

Next, we analyzed the effects of the various toxic and non-toxic human disease proteins upon mitochondrial distribution, using the mito-HA-GFP marker (mitoGFP), a fusion between the 31-amino-acid mitochondrial import sequence from human cytochrome c oxidase subunit VIII fused and the N-terminus of GFP (Pilling et al., 2006). Several studies have used this marker in the *Drosophila* system and found effects of human neurodegenerative disease proteins upon mitochondrial structure and distribution (Deng et al., 2008; Yun et al., 2008; Iijima-Ando et al., 2009; Park et al., 2009; DuBoff et al., 2012; Klein et al., 2014; Mhatre et al., 2014).

**Fig. 5.**
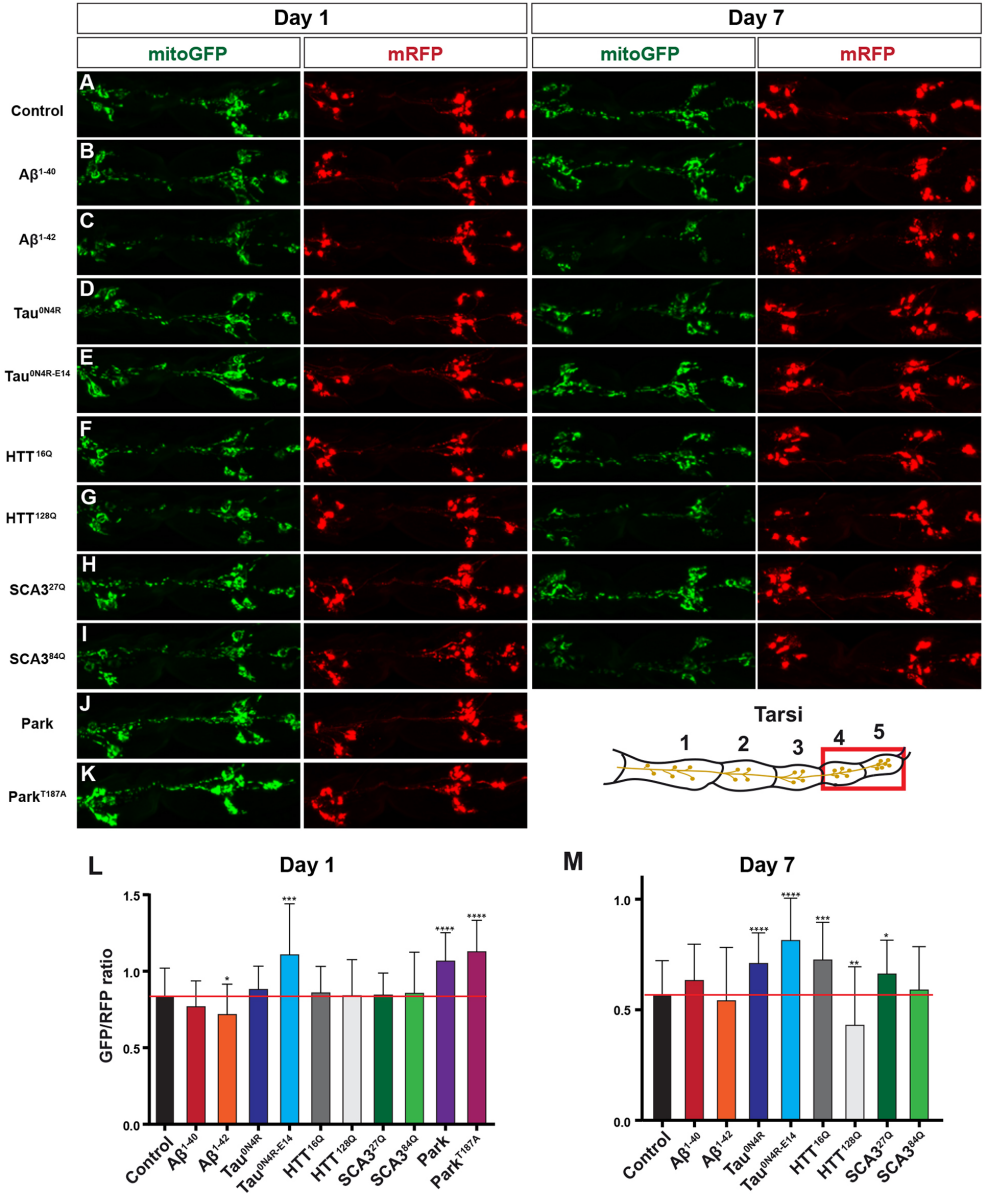
**Human neurodegenerative disease proteins affect mitochondrial distribution in fly leg sensory cell bodies.** (A-K) Control (*attP65B2*) and *UAS* lines were crossed to *OK371-Gal4*, *UAS-mitoGFP;UAS-mRFP*, to direct expression to glutamatergic neurons in the fly leg and to enable analysis of mitochondrial distribution. Panels show representative confocal images of projected sections through tarsi 4-5, on day 1 and day 7, at +29°C. (L,M) Quantification of the mitoGFP and mRFP levels in sensory neuron cell bodies in tarsi 4-5, at day 1 and day 7. Graph shows the ratio of mitoGFP signal over mRFP signal for each measured cell body. Tau^0N4R-E14^, Park and Park^T187A^ showed a significant increase in mitoGFP/mRFP ratio when compared with the control at day 1. In addition to those, on day 7 also Tau^0N4R^ and both the shorter versions of the poly-Q repeat proteins, HTT^16Q^ and Sca^27Q^, showed an increase in mitoGFP/mRFP ratio. A reduction in the mitoGFP/mRFP ratio was detected only in Aβ^1-42^ on day 1, but this reduction was lost at day 7. The longer repeat of HTT (HTT^128Q^) showed a reduction on day 7. Other disease proteins did not display a striking effect upon the mitoGFP/mRFP ratio. Owing to the reduction in signal in deeper layers, only cells immediately under the cuticle were analyzed (*n*≤26 cells, *n*≤6 legs; mean+s.d.; *P≤0.05; **P≤0.01; ***P≤0.001; ****P≤0.0001; Student's two-tailed *t*-test, pair-wise against control).

We combined *UAS-mitoGFP*, *UAS-myr-mRFP* and *OK371-Gal4*, in order to visualize both mitochondria and the entire neuronal cell bodies simultaneously. First, we analyzed the distribution of mitochondria in the sensory neuron cell bodies in tarsi 4-5. In control flies, at both day 1 and day 7, we observed a robust mitoGFP signal in the cell body (Fig. 5A). Turning to the disease proteins, we observed an apparent increase in mitoGFP signal in some strains (Tau^0N4R-E14^, Tau^0N4R^, Park, Park^T187A^, HTT^16Q^ and SCA3^27Q^) and a reduction of mitoGFP signal in others (Aβ^1-40^, Aβ^1-42^, HTT^128Q^ and SCA3^84Q^; Fig. S2A,B). Other disease proteins did not display significant effects upon the mitoGFP signal (Fig. S2A,B). To uncouple the change in intensity of mitoGFP in the cell body from a possible general sickness of the cell, we also measured the mRFP levels (Fig. S2C,D) and plotted the ratio of mitoGFP to mRFP (Fig. 5L,M). This revealed a significant increase in mitoGFP/mRFP ratio in Tau^0N4R-E14^, apparent when comparing both with control and with Tau^0N4R^, at both day 1 and day 7 (Fig. 5A,D,E,L,M). In addition, Tau^0N4R^ also showed a significant increase in mitoGFP/mRFP ratio on day 7. Likewise, expression of Park or Park^T187A^ caused an increase in mitoGFP/mRFP ratio at day 1, and interestingly, Park^T187A^ showed stronger effects than Park, correlating with the overall toxicity seen in the lifespan experiments (Fig. 5A,J-M; owing to lethality, we could not test Park^T187A^ at day 7). The expression of the shorter version of the poly-Q repeat protein, HTT^16Q^, showed an increase, whereas the longer version, HTT^128Q^, showed a decrease in the mitoGFP/mRFP ratio at day 7 (Fig. 5A,F,G,L,M).

**Fig. 6.**
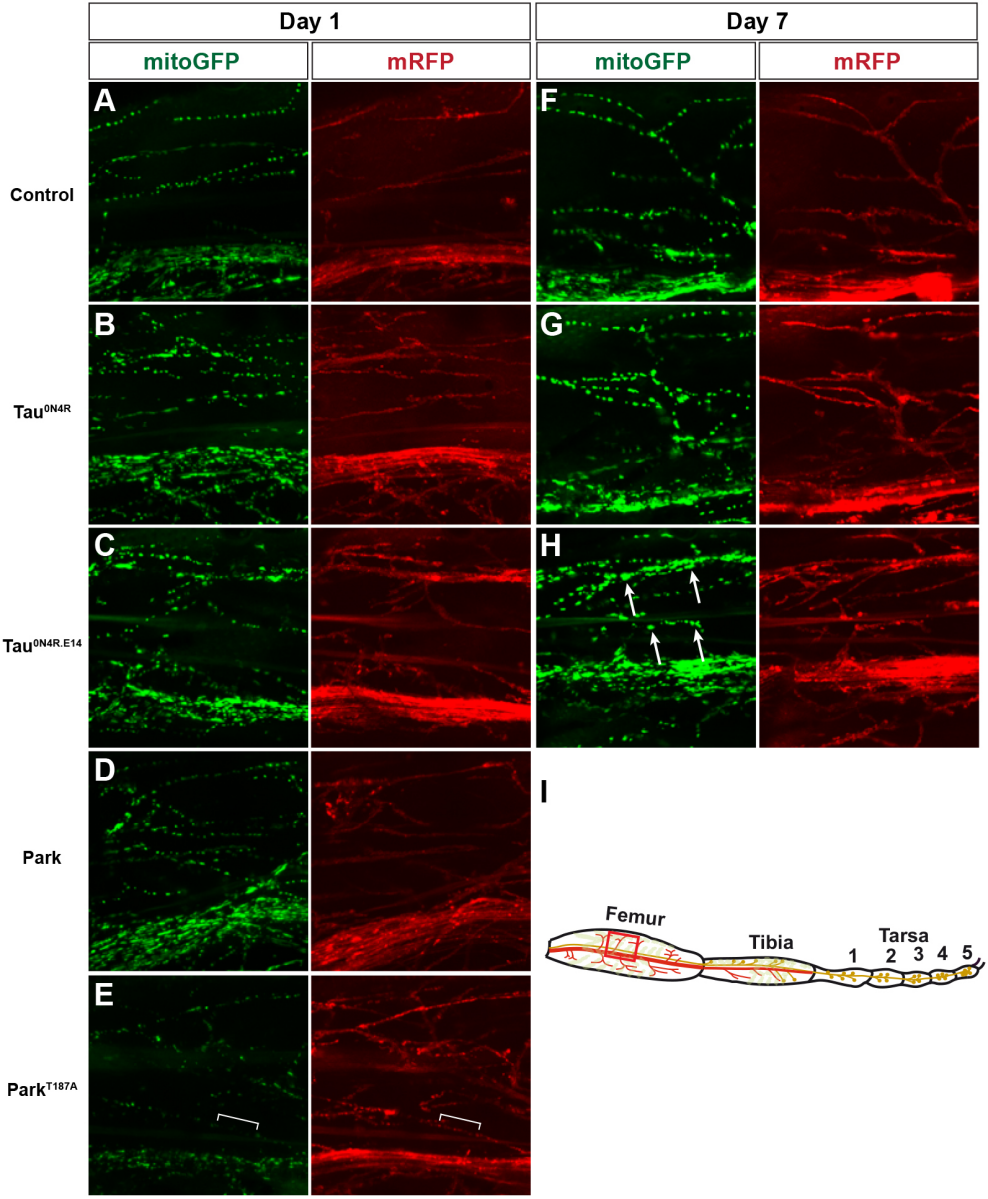
**Human neurodegenerative disease proteins affect mitochondrial distribution in fly leg motor neuron axonal terminals.** (A-H) Control (*attP65B2*) and *UAS* lines were crossed to *OK371-Gal4*, *UAS-mitoGFP;UAS-mRFP* to direct expression to glutamatergic motor neurons innervating the fly leg femur and to enable analysis of mitochondrial distribution. Panels show representative confocal images, for the indicated transgenic lines, of projected sections through a femur region (red box in I) after day 1 and day 7, at +29°C. In control, mitochondria are evenly dispersed along axons tracts and show similar morphology. In Tau^0N4R^ and Tau^0N4R-E14^, mitochondria have irregular shapes, and clumps form in the axons, in particular at day 7 (arrows in H). In Park^T187A^, gaps free of mitochondria are evident in axons (brackets in E).

Next, we turned to the distribution of mitochondria in the femur, focusing on the motor neuron terminal projections into the muscles. In control flies, we observed an even distribution of mitochondria along the terminal projections, with similar appearance at day 1 and day 7 (Fig. 6A,F). When expressing the disease proteins, we observed an apparent ‘clump-like’ aggregation of mitochondria in both Tau^0N4R^ and Tau^0N4R-E14^, being most pronounced in the latter, and increasing in severity from day 1 to day 7 (Fig. 6B-C,G,H). By contrast, Park and Park^T187A^ showed a severe reduction in the number of mitochondria present in the terminal, with the latter being more pronounced (Fig. 6D,E). Aβ^1-42^ also displayed a striking reduction in mitochondria in the motor terminals (Fig. S1C,L). Other disease proteins did not display striking effects upon mitoGFP expression or localization (Fig. S1). To quantify the observed effects in the axons, we counted axon sections of at least 20 µm without mitochondria. We identified two to four axons with such gaps in each confocal femur scan of Park^T187A^, but none in the other genotypes (Table S2). In summary, there is a variety of effects on mitochondrial distribution and dynamics observed using mitoGFP in the adult fly leg neurons, with the most striking effects seen when expressing the mutant versions of Tau (Tau^0N4R-E14^) and Park (Park^T187A^).

## DISCUSSION

### Correlation between toxicity effects when comparing lifespan, geotaxis and cell survival

For the majority of human disease proteins tested in this study, we find good agreement between their organismal toxicity, as revealed by lifespan and geotaxis assays, on the one hand, and cell toxicity, as revealed by complete loss of nGFP expression, on the other. For instance, Aβ^1-42^ and HTT^128Q^ both severely affect lifespan and geotaxis and also show striking loss of nGFP-expressing cells at day 10-14, with a loss of some two-thirds of nGFP-expressing cells. Interestingly, however, Park^T187A^ and ATX1^82Q^, which are the most toxic strains with respect to lifespan and geotaxis (Park^T187A^), did not show any loss of nGFP-expressing cells at day 1, a mere day before all flies had died. Likewise, Tau^0N4R-E14^, which showed an average lifespan of only 8 days, did not show any effects on the number of nGFP-expressing cells even at day 10-14. Although we cannot confirm from this experiment that the gradual, then final loss of the nGFP signal in these sensory cells in adult legs of Aβ^1-42^, HTT^128Q^ and SCA3^84Q^ flies is the result of cell death, we believe that this is a strong indicator of cell death. Our results from expressing human ND proteins are in general agreement with previous studies with regard to lifespan and geotaxis. For example, although expression of SOD1^G85R^ mutant protein resulted in no adverse effects on lifespan, the flies still showed impaired locomotor function (Fig. 1), as previously shown (Watson et al., 2008).

### F-actin structures are affected by expression of most neurodegenerative disease proteins

The use of Lifeact-Ruby to label F-actin processes revealed the presence of actin filament processes in the immediate axon emanating from the sensory cell bodies (Fig. 4A). Interestingly, this Lifeact-Ruby labeling is reminiscent to that of labeling of the vertebrate AIS (Jones and Svitkina, 2016). Vertebrate AIS contains microtubules coated with a dense protein network of Ankyrin G, βIV-spectrin and F-actin (Palay et al., 1968; Watanabe et al., 2012; Xu et al., 2013; Jones et al., 2014; Eira et al., 2016). The role of the AIS includes a site for action potential firing and for maintaining neuronal polarity (Jones and Svitkina, 2016). Its cytoskeletal part acts as a screening filter for vesicle trafficking by regulating axonal entry and exit of cargos. Interestingly, perturbation of the AIS cytoskeleton has recently been observed in ND, such as AD (Sun et al., 2014; Tsushima et al., 2015). It has been debated whether *Drosophila* neurons contain such a segment (Rolls, 2011). However, recent studies revealed that *Drosophila* Ankyrin, Ank2, is a conserved molecule acting as an axonal diffusion barrier, indicating the presence of an AIS structure also in *Drosophila* (Jegla et al., 2016).

Intriguingly, we found a complex relationship between organismal toxicity and F-actin scaffold integrity in the sensory cell bodies and the immediate axon. Specifically, several proteins with high organismal toxicity, evident by short lifespan and impaired geotaxis, did indeed show severe effects on Lifeact-Ruby. These include Aβ^1-42^ and ATX1^82Q^, both of which strongly affect both lifespan/geotaxis and Lifeact-Ruby labeling. By contrast, Park^T187A^ and HTT^128Q^, in spite of being highly toxic in the adult fly, did not show striking effects on Lifeact-Ruby labeling. Interestingly, both Tau^0N4R^ and Tau^0N4R-E14^ showed strong effects upon Lifeact-Ruby labeling. In line with these results, the role of tau has recently been expanded from regulating microtubule stability to also regulating the actin cytoskeleton, and studies suggest a causative role between tau pathology and F-actin stabilization (Moraga et al., 1993; Farias et al., 2002; Fulga et al., 2007; He et al., 2009; DuBoff et al., 2012; Frost et al., 2014, 2016; Elie et al., 2015).

Furthermore, loss of polarized distribution or mis-sorting of pathogenic tau from the axons to the somatodendritic compartments is a key early event in diseases such as AD and frontotemporal dementia with parkinsonism linked to chromosome 17 (Zempel and Mandelkow, 2014). Hence, it is tempting to speculate that the loss of Lifeact-Ruby labeling observed in our study reflects defective AIS-like structures. Future studies, analyzing the presence of Ankyrins in this segment of the leg sensory neurons, might help to reveal whether the AIS barrier is disrupted, in which case the toxicity could be attributable to erroneous transport of cargo, or indeed tau itself, into somatodendritic compartments. In fact, mis-sorting of tau through pathogenic acetylation (Sohn et al., 2016) or mis-sorting of tau as a result of Aβ^1-42^ insult (Zempel and Mandelkow, 2012) was previously shown to compromise the AIS compartment. The increasingly strong link between cytoskeletal impairments and ND raises the potential for new therapeutic strategies (Eira et al., 2016). The straightforward analysis of leg sensory neurons using Lifeact-Ruby described here might provide an interesting *in vivo* read-out for future drug screening aimed at targeting cytoskeletal impairments.

### ND proteins affect mitochondrial distribution, integrity, or both

To address the effects of ND proteins upon mitochondrial integrity, we coexpressed the marker mitoGFP together with mRFP. Focusing first on the cell bodies, we compared the ratio of mitoGFP to mRFP levels, in order to avoid erroneous interpretations based solely on mitoGFP. We observed a significant reduction in the mitoGFP/mRFP ratio in HTT^128Q^ on day 7 and in Aβ^1-42^ on day 1. The reduction in the mitoGFP/mRFP ratio for Aβ^1-42^ was no longer seen on day 7, although both mitoGFP and mRFP levels were reduced, implying that cells were dying and thus losing both signals. Huntingtin has a widely established role in axonal transport, for example of mitochondria. However, it has been debated whether the pathology in HD arises because of loss of function or indeed from toxic gain-of-function effects of the expanded poly-Q repeat (Gunawardena et al., 2003; Lee et al., 2004; Schulte and Littleton, 2011). Our results suggest problems with axonal transport of mitochondria, but we cannot exclude the possibility that the toxicity of HTT^128Q^ is attributable to other cytotoxic events.

In contrast to the reduction in mitoGFP/mRFP ratio in HTT^128Q^ and Aβ^1-42^, we observed an increased ratio in HTT^16Q^, SCA^27Q^, Park, Park^T187A^ and Tau^0N4R-E14^, indicating defects in mitochondrial transport, dynamics or morphology. Given that the mitoGFP signal in the cell body was enhanced compared with control, and there was no significant increase in the mRFP signal, this suggests that the effect is not attributable to mere changes in UAS-expression levels. The effects were more severe in Tau^0N4R-E14^ than in Tau^0N4R^, suggesting involvement of tau phosphorylation, but Tau^0N4R^ also showed an effect as the flies aged (Fig. 5L,M). Both tau and parkin have been shown to be involved in mitochondrial fission, and thus these effects might be a reflection of mitochondrial morphogenesis. In line with this argument, DuBoff et al. (2012) have shown that expression of tau in *Drosophila* neurons gives rise to elongated mitochondria, where the severity of morphology is correlated with neurotoxicity and is enhanced with age, in addition to being enhanced in the more toxic Tau^E14^ form.

Interestingly, in both the shorter versions of HTT and SCA3 (HTT^16Q^ and SCA3^27Q^), we saw an increased mitoGFP signal in cell bodies, whereas in the longer version (HTT^128Q^ and SCA3^84Q^) it was decreased (Fig. S2). As only the longer version of HTT gave rise to organismal toxicity and premature death, we conclude that the loss of mitoGFP/mRFP signal in cell bodies that arose as the flies aged correlates with this and could reflect a defect in mitochondrial transport, biogenesis, or both, in line with previously published data (Reddy and Shirendeb, 2012). In fact, evidence suggests that defects in mitochondrial biogenesis are also a contributing factor in HD (Reddy, 2014), and addressing the defect in mitochondrial fission and fusion is emerging as a new therapeutic target.

Looking at the axons, we noted an interesting difference between Tau^0N4R-E14^ and Park^T187A^. Although both displayed an increase of mitoGFP in the cell body, Park^T187A^ showed a severe reduction of mitochondria in axon terminals, whereas Tau^0N4R-E14^ showed an apparent accumulation or clumping of mitochondria in the terminals. It is tempting to speculate that this might reveal fundamental differences in their effects upon axon transport, mitochondrial integrity, or both. Park and PINK1 kinase play a well-established role in the quality control of mitochondria, regulated via mitochondrial fission and fusion dynamics (Pickrell and Youle, 2015). In Park^T187A^, T187 represents a site phosphorylated by PINK1 kinase in humans; hence, the alanine mutation inhibits this phosphorylation. Phosphorylation of Park by PINK1 has been described as important for Park localization to mitochondria (Kim et al., 2008), thus we suggest that the toxicity and mobility defects in Park^T187A^-expressing flies would be attributable to inappropriate mitochondrial control. We conclude that there are protein-specific effects upon mitochondrial distribution and integrity, which do not always match the toxicity effects apparent in lifespan and geotaxis assays, because of the underlying dominant function of the ND protein.

### Developing a ‘bar-coding’ system for proteotoxicity

**Fig. 7.**
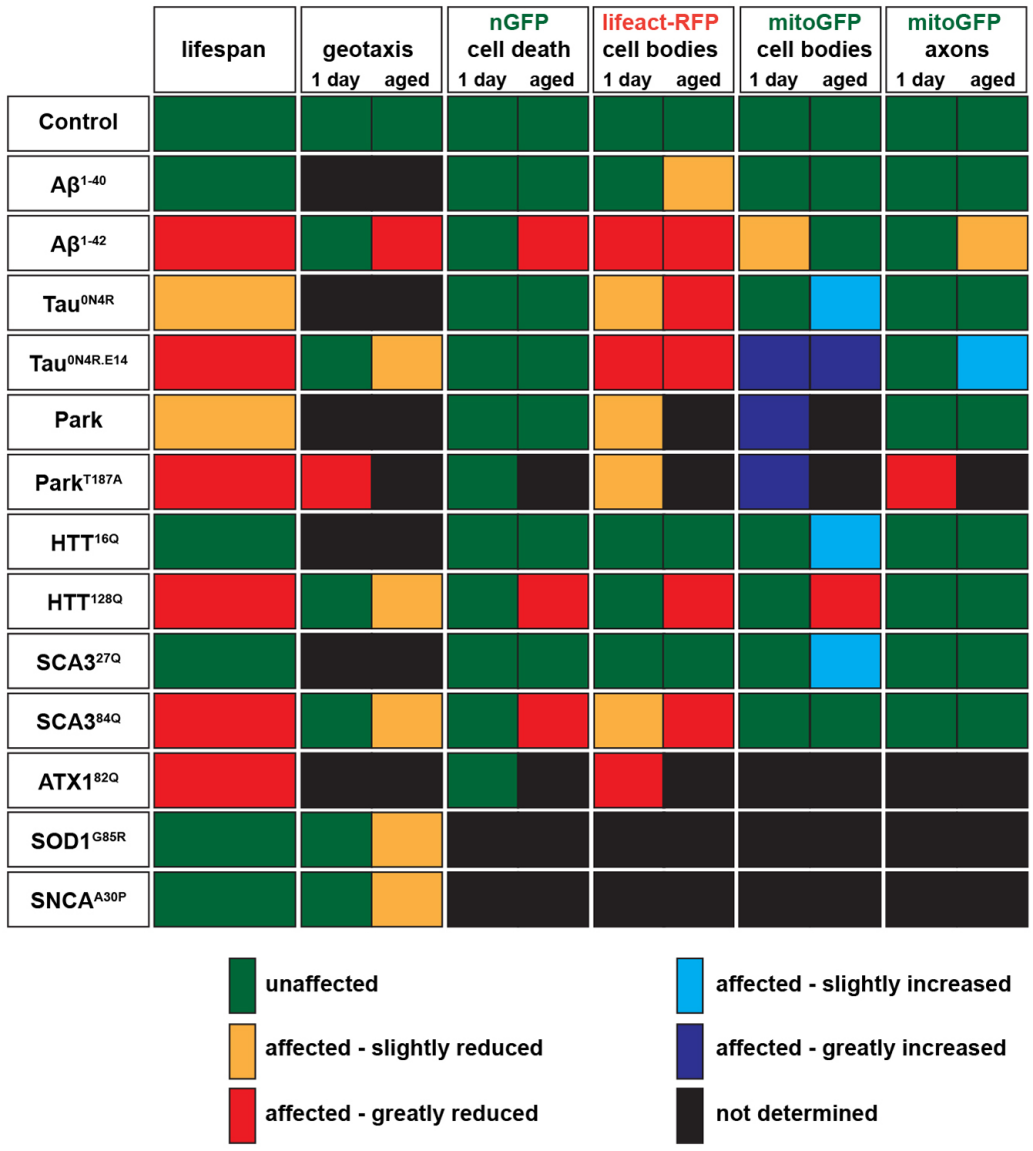
**Bar-coding neurodegeneration.** Summary of observed effects of human disease proteins when expressed in *Drosophila* leg neurons using the *OK371-Gal4* driver. See text for details.

Summarizing the effects upon the various read-outs and markers used in this study, in a simple ‘bar-coding’ scheme, we can observe a clear divergence in the various proteotoxic effects when comparing the different human disease proteins with each other (Fig. 7). This might be somewhat surprising, because a number of studies have pointed to general and common toxicity mechanisms of the many misfolding and aggregating ND disease proteins (Han and Shi, 2016; Weishaupt et al., 2016; Ahmad et al., 2017; Islam, 2017; Krench and Littleton, 2017; Lin et al., 2017). However, the proteins studied here are different from each other with respect to their protein structure and their normal cellular functions. On that note, it is perhaps not surprising that toxicity analysis using multiple markers and assays uncovers protein-specific effects. We would envision that further development of adult leg neuron fluorescent reporters might help to develop this system into a powerful high-throughput assay for distinct cellular mechanisms of human ND disease protein toxicity.

## MATERIALS AND METHODS

### Fly stocks

*UAS-Aβ_1-40_*, *UAS-Aβ_1-42_* and *n-Syb-Gal4* were previously described (Jonson et al., 2015). *UAS-Tau^0N4R^* was created by site-specific integration at the 53B site on chromosome 2 (BestGene) (Fernius et al., 2017). *UAS-Tau^0N4R-E14^* was kindly provided by Amritpal Mudher (Southampton, UK); *UAS-nmGFP* (Allan et al., 2003). Other *UAS-GFP/RFP* reporter transgenes were obtained from Bloomington Stock Center and are listed in Table S1.

Other strains obtained from Bloomington Stock Center were as follows: BL#9750, *attP65B2*; BL#33808, *UAS-HTT^128Q^*; BL#33810, *UAS-HTT^16Q^*; BL#33610, *UAS-SCA3^84Q^*; BL#33609, *UAS-SCA3^27Q^*; BL#33818, *UAS-ATX1^82Q^*; BL#33608, *UAS-SOD1^G85R^*; BL#8147, *UAS-SNCA^A30P^*; BL#34748, *UAS-PARK^T187A^*; BL#51651, *UAS-PARK*; and BL#26160, *OK371-Gal4*.

### Lifespan assay

Flies were kept at +25°C at 60% humidity, under a 12 h:12 h light:dark cycle until eclosion, and at +29°C after eclosion. Crossings were reared in 50 ml vials with standard *Drosophila* food (corn meal, molasses, yeast and agar). Newly eclosed flies were maintained at +29°C in 50 ml vials containing rich *Drosophila* food (water, potato mash powder, corn flour, yeast, agar, syrup, propionic acid (diluted: 48.5 ml propionic acid+∼950 ml H_2_O) and green food coloring]. Every 2-3 days, flies were transferred to fresh vials, and surviving flies were scored. GraphPad Prism 6.0a software (GraphPad Software) was used to generate Kaplan–Meier survival curves (Kaplan and Meier, 1958).

### Negative geotaxis assay

Transgenic *UAS* flies were crossed to the *OK371-Gal4* line and kept at +26°C until eclosion. The female flies were sorted and placed in ten vials with ten flies per vial, and placed at +29°C. Flies were examined on day 1 and on day 10-14, to assess the viability of all transgenic flies over this time range. For *UAS-Tau^0N4R-E14^*, male flies were used instead of females because low numbers of female flies hatched. Flies were always allowed to recover from CO_2_ for at least 3 h until assayed. Flies were flipped into new, empty vials and allowed to acclimate for 30 s before starting the assay. Flies were gently shaken to the bottom of the vial, and the percentage of flies that climbed up to a 5 cm mark on the vial within 30 s was counted, and the procedure was repeated ten times for each vial. The mean with s.d. is plotted.

### Preparation of adult fly legs for microscopy

Adult front legs were removed with scissors and placed on a microscope slide. Ten microliters of mounting medium (DABCO/PVP) was added and a cover glass placed on top.

### Confocal imaging and data acquisition

A Zeiss LSM 700 confocal microscope was used for fluorescent images; confocal stacks were merged using LSM software or Adobe Photoshop. Statistical calculations and Kaplan–Meier survival curves (Kaplan and Meier, 1958) were performed in GraphPad Prism software (v.4.03). Images and graphs were compiled in Adobe Illustrator.

### Assessment of intracellular markers in cell bodies and axons

Transgenic *UAS* flies were crossed with fly strains carrying intracellular markers and *OK371-Gal4* and kept at +26°C until eclosion. Flies were kept overnight at +29˚C and analyzed on day 1 and on day 7, where possible. *UAS-ATX1^82Q^* flies were crossed at +20°C to enable viable offspring to hatch, after which they were transferred to +29°C.
